# Letter to editor about “Development and validation of a machine learning-based risk model for metastatic disease in nmCRPC patients: a tumor marker prognostic study”

**DOI:** 10.1097/JS9.0000000000003414

**Published:** 2025-09-10

**Authors:** Liuhai Zeng, Wei Lin, Haiyan Wu

**Affiliations:** Department of General Surgery, Xiangshan Hospital of Wenzhou Medical University, Ningbo, Zhejiang, P.R. China

*Dear Editor,*

We read with great interest the recent article published in your journal entitled “Development and validation of a machine learning-based risk model for metastatic disease in nmCRPC patients: a tumor marker prognostic study”^[1]^. Drawing on data from two phase III clinical trials, SPARTAN and ARAMIS, the authors developed and validated a prognostic model that offers new insights into risk stratification and personalized management of nmCRPC. While we commend the authors for leveraging high-quality RCT data to construct a multivariable predictive tool, we would like to raise several points for further discussion and consideration. This letter to the editor conforms to the TITAN Guidelines 2025 on authorship statements and AI usage^[2]^.

First, the model incorporated eight predictors, including treatment type, Gleason score, prior therapy, PSADT, log PSA, and hemoglobin, all of which are clinically relevant. However, some elements warrant closer scrutiny. The inclusion of “race (White)” as a positive risk factor (HR = 1.23) may reflect confounding factors such as healthcare access and regional socioeconomic status, rather than intrinsic biological differences^[3]^. Given the model’s intended application across diverse populations, we suggest reevaluating this variable or considering alternative adjustments. The designation of prior local therapy (surgery + radiation) as a high-risk marker, though theoretically plausible due to tumor selection pressure and clonal evolution, could also reflect a “reverse causality” bias, wherein higher-risk patients are more likely to receive aggressive treatment. This warrants cautious interpretation. Additionally, while hemoglobin was included as a prognostic factor, it lacks specificity for metastasis and may instead serve as a proxy for nutritional or comorbid conditions^[4]^; its independent clinical value remains uncertain.

Second, with the increasing integration of molecular imaging techniques such as PSMA PET into clinical practice, studies have revealed that nearly 50% of nmCRPC patients deemed metastasis-free by conventional imaging may harbor “occult metastases.” Although the authors acknowledged the ongoing debate around PSMA PET and its biological implications, and noted its current limitations in replacing existing stratification standards, we believe future predictive models should aim to integrate traditional clinical parameters with molecular imaging features. While the present model retains practical value in imaging-negative populations, further work is needed to harmonize such tools with advanced imaging findings for more accurate risk prediction.

Lastly, while we recognize the model’s utility in clinical trial stratification – particularly in guiding follow-up intensity and treatment escalation for high-risk patients – it is important to note that both model construction and validation were based on RCT populations. The lack of validation in independent real-world cohorts limits its generalizability. Prospective evaluation in large, multicenter registry studies would strengthen the evidence base for its broader application.

In conclusion, this study represents an important step forward in addressing the unmet need for risk prediction in nmCRPC. The proposed model and stratification framework offer valuable reference points. However, we encourage further refinement of variable selection, validation in more heterogeneous populations, and integration with novel molecular imaging modalities to develop a more robust, generalizable multimodal predictive tool. Such efforts will be essential to advancing personalized management in nmCRPC.

## Data Availability

Any datasets that were not generated and/or analyzed during the course of this study.

## Research registration unique identifying number (UIN)

Not involving human subjects.
